# A Contextual Behavioral Account of Culture: Example Implementation of a Functional Behavioral Approach to the Study of Cultural Differences in Social Anxiety

**DOI:** 10.3389/fpsyg.2020.00418

**Published:** 2020-03-10

**Authors:** Alexander Krieg

**Affiliations:** Department of Global Communication, Kobe Gakuin University, Kobe, Japan

**Keywords:** culture, functional behavioral assessment, contextual behavior science, social anxiety, cross-cultural differences

## Abstract

The current article proposes integrating a functional behavior approach to the study of culture. After describing culture from a contextual behavioral science framework, we outline a three-step process to perform a functional behavior analysis of culture: (1) identifying potential contingencies, (2) determining functional relationships, and (3) gathering supporting evidence. As an example, we present each of the three steps through a re-analysis of data related to cultural differences in social anxiety between Japanese and European Americans as well as describe a hypothetical experiment. The results demonstrate how implementing an alternative framework that focuses on the relationship between behavioral function and environmental adaptability leads to different conclusions compared to implementing frameworks that emphasize the form or degree of a behavior or belief in one group compared to another. For this particular example, in contrast to viewing social anxiety in Japanese as something stemming from innate beliefs about themselves and others (e.g., self-construal), the current study suggests that displaying social anxiety in some situations within a Japanese context is more functionally adaptive (e.g., more likely leads to desirable outcomes) than within a European American context.

## Introduction

Contextual Behavior Science (CBS) is the functional analysis of behavioral variation, selection, and retention within a given context across various dimensions (Hayes et al., 2012, 2017; Zettle et al., 2016). One important contextual dimension is culture. Culture has been defined by cultural psychologists as a set of shared values, beliefs, and practices that are influenced by the environment and transmitted to others (Kitayama and Cohen, 2007; Markus and Hamedani, 2007). According to this widely agreed-upon definition, culture characterizes both an individual’s private thoughts (i.e., values and beliefs) and public behaviors (i.e., practices) *if* they are (1) influenced by salient features of the physical or social ecology, (2) shared by a specific group, and (3) transmitted by the group to new culture members.

Contextual Behavior Science focuses on predicting and influencing both public behavior (practices) as well as private internal behavioral events (thoughts, feelings, and values), and provides a compelling account for how these behaviors are shaped by contextual factors. Specifically, CBS addresses (1) how the features of the environment create the contingencies that promote or obstruct a behavior from occurring (e.g., behavioral antecedents), (2) how social learning can occur so that individuals in close proximity share similar private and public behavior (e.g., behavioral imitation, Epstein, 1984; Boyd and Richerson, 1985), and (3) how behavioral repertoires are transmitted to others via the processes of associative and operant learning (e.g., behavioral consequences). Taken together, CBS aims to functionally relate organisms’ behaviors with features of the environment.

Many contributions to the study of culture emphasize the form, frequency, or intensity of a certain groups’ behavior relative to other groups. Unfortunately, the relation between that groups’ behavior and the environment in which they are situated is often relegated to the discussion section of a manuscript if addressed at all (Ryder and Chentsova-Dutton, 2015). In contrast, CBS emphasizes function over form, highlighting the role of environmental contingencies (i.e., antecedents and consequences). To a behaviorist, the question is not limited to just “how much more frequent does this behavior need to occur to be representative of a given culture?” but rather “is this behavior being differentially reinforced by even a subsection of the population?” (Tourinho and Vichi, 2012; Glenn et al., 2016; Baum, 2017). If it is, that alone creates a sufficient basis for cultural inquiry.

The current study provides a brief CBS account of culture, as well as re-analyzes the results of a recent study on Japanese and European American differences in social anxiety (Krieg, 2018) from a CBS perspective. In doing so, we hope to provide a theoretical framework for the contextual influence of culture on individual behavior as well as expound a CBS-approach for future studies to examine and compare cultures beyond the form and frequency of cultural practices or beliefs such as “individualism and collectivism.”

### Contextual Behavioral Science

Contextual Behavior Science claims academic heritage from Charles Darwin, B. F. Skinner, and Murray Sidman, all of whom emphasized the role of environment × organism interactions in the variation, selection, and retention of a given behavioral repertoire (Hayes et al., 2017). Rooted the philosophy of functional contextualism, CBS emphasizes the centrality of situated action and sets a pragmatic truth criterion, attempting to answer the question “what works in this context” (Hayes et al., 2012). It utilizes various behavior analysis methodology to develop a basic behavioral account for complex organism behavior (Blackledge, 2003).

Behavior variation occurs somewhat randomly (Hayes et al., 2017), with the environment simply setting constraints on what behavior is possible in a certain context (e.g., singing is impossible for humans to perform underwater). From a pool of possible behaviors, the environment influences the selection and retention of behavior either by generalizing a behavioral response across a variety of stimuli through associations (i.e., classical conditioning; Pavlov, 1902) or through providing appetitive or aversive outcomes toward a behavior exhibited in a certain context (i.e., operant conditioning; Skinner, 1938, 1963).

Several interventions were developed from CBS and have shown a high degree of clinical utility across a broad range of affective and behavioral concerns. For example, Acceptance Commitment Therapy (ACT) is a modern, process-based contextual behavior therapy that has demonstrated effectiveness in treating a wide range of psychological phenomena all over the world (Hayes et al., 2006; Powers et al., 2009; A-tjak et al., 2015). Similarly, Applied Behavior Analysis (ABA) is the gold standard treatment for treating children with developmental disabilities worldwide (e.g., Austin and Carr, 2000; Hayes and Bissett, 2000). Making use of its sensitivity to subtle idiographic factors impacting an individual’s behavior in a specific context, several attempts have been made to capitalize on the potential for cultural sensitivity it offers (Hayes et al., 2011; Pasillas and Masuda, 2014; Sabucedo, 2017). These approaches to working with psychological and behavioral concerns have become invaluable tools within the modern clinical psychologist’s toolbox.

### Contextual Behavioral Ideas in Cultural Psychology

A lesser-known fact among non-behaviorists is that Skinner’s treatment of human behavior inherently stressed the importance of social and cultural variables (Skinner, 1981). This idea is so central to a behavioral understanding of culture that Skinner (1953) even defined culture as contingencies “arranged by other people” (p. 419). Specifically, Skinner (Skinner, 1953; Ferster and Skinner, 1957) hypothesized that an individual’s behavior often constitutes a significant portion of the controlling environment for the behavior of other individuals and defined social behavior on the basis of these “interlocking behavioral contingencies” (IBCs). According to the theory, IBCs may give rise to cultural practices when the behaviors involved are learned by other individuals and maintained by similar contingencies (Muchon de Melo and de Rose, 2013). Taken together, the behavior of other group members provides the antecedents and consequences of cultural practices.

The idea of understanding culture in terms of its antecedents and consequences is not new to the field of cultural psychology. Although a simplification, it could be said that traditional culture-comparative research defined culture as a collection of antecedent conditions that shaped behavior. If some difference in behavior was observed between two groups there should be an antecedent variable that is able to account for this. The presumption of invariant antecedent-behavior relationships across human populations in all cultures was reflected by the notion of universalism, a prevalent belief at the time (Triandis, 2007).

However, as the assumption of the universality of humankind gave way to the apt criticism offered by the indigenous and cultural psychology traditions (Stigler et al., 1990; Sinha, 1997), this approach fell out of favor and more mentalistic concepts such as individualism and collectivism (Triandis, 1995) and self-construal (Markus and Kitayama, 1991) became increasingly popular. Although these concepts demonstrated some utility in certain explanatory models, there are certain philosophical challenges associated with “invisible” mentalistic concepts. As outlined by Ryle (1984), logical problems occur when “categories of behavior” are conflated with the behaviors themselves. In his famous example of ‘team spirit,’ we can observe teammates shouting, patting, and hugging each other after winning a sporting event, but there is no “ghostly team spirit” running around the field or possessing each of the players (Baum, 2017). Perhaps unsurprisingly, methodological limitations followed this shift toward mentalistic concepts, as cultural scientists struggled to define, measure, and support cultural validity/equivalence for these new mentalistic constructs (e.g., Levine et al., 2003). The current paper’s author is reminded of an interaction he witnessed at the International Association of Cross-Cultural Psychology’s (IACCP) 2016 Conference in Nagoya, Japan where a prominent cultural psychologist asked a room full of conference attendees “No, really, we talk about self-construal, but do we even know what a ‘self’ is?” The room went silent.

It is possible that the de-emphasis of behavioral antecedents and consequences from the study of cultural psychology was a little premature. Recent developments in both CBS as well as evolutionary branches of cultural psychology may have made a way for more promising collaborations between the two fields. Whereas the methodological behaviorism of the 1960’s–1980’s offered a less than convincing account of verbal behavior as well as individuals’ internal thoughts or feelings (Chomsky, 1959; Winton, 1986), advances in theories such as Relational Frame Theory (RFT; Hayes et al., 2001) have opened up verbal behavior and “private internal behavioral events” (i.e., thoughts and feelings) as not only legitimate but fruitful topics of inquiry using modern behavioral methodology. Likewise, in cultural psychology, there seems to be a resurgence of the notion that the physical and social environment can profoundly affect the phylogenic development of people groups both on genetic and cultural levels (Cole and Hatano, 2007; Konner, 2007; Newson et al., 2007; Keller, 2008).

Recent research on gene-culture coevolution (e.g., Boyd and Richerson, 1985; Laland et al., 2010) and cultural neuroscience (e.g., Chiao et al., 2008, 2013; Han and Humphreys, 2016) has supported the integration of natural and cultural science and emphasized the dynamic organism × environment interactions on both the individual and culture group levels (Kashima, 2014, 2016). Likewise, the modern CBS approach has increasingly integrated within a larger framework of evolutionary science (Hayes et al., 2012, 2017; Wilson et al., 2018), and wholeheartedly agrees with these propositions.

### A CBS Approach to Examining Culture

As discussed in previous sections, a functional behavioral approach to culture emphasizes identifying and understanding the contingencies of a given behavior rather than overly focusing on the behavior itself. The goal is to understand the resulting function of a given behavior and how it impacts the individual or the individual’s environment. This is usually described as the ABC’s of behavior analysis (Sturmey, 1996; Iwata, 2000), where behaviors are defined by their functions in the following format.

[Behavior] functions to [Consequence] when/among/during [Antecedent]

*Example:* [Nodding one’s head] functions to [keep a speaker talking] during [conversation.]

In order to identify the components necessary to complete the above conclusion, the current paper recommends the following three-step process: (1) identifying potential contingencies, (2) determining functional relationships, and (3) gathering supporting evidence. These three steps are discussed in turn in the sections below.

#### Step 1: Identifying Potential Contingencies

In order to develop a list of potential contingencies that may account for a specific behavior, researchers can ask questions related to the behavior’s variation, selection, and retention to better elucidate these exact mechanisms. Table 1 outlines a non-exhaustive list of possible questions. The first set of questions relates to defining the research question and behavior of interest in concrete behavioral terms. This is in order to avoid the difficult task of attempting to use environmental contingencies to predict an entirely mentalistic concept. Next, it is recommended that the researcher takes a moment to identify alternatives to their behavior of interest. If other behaviors are *not* occurring in this context or some do at much lower base rates, this information is likely beneficial in forming hypotheses about possible antecedents and consequences.

**TABLE 1:** Questions to facilitate the identification of potential cultural contingencies on behavior.

Process	Question
Behavioral definition	How would one define the current object of study in behavioral terms?
	Is it possible to divide this behavior into smaller units?
Variation	What alternative behaviors are possible?
	What relevant behaviors are occurring at a lower base rate?
	What relevant behaviors are not occurring at all?
Selection antecedents	In what social/physical ecology does this occur?
	Are there social/physical ecologies in which this does not occur?
	Does this social/physical ecology occur in other culture groups’ set of social/physical ecologies? If so, do similar behaviors emerge?
	Are there any features in the social/physical ecology that are sufficiently salient to be a potential stimulus control variable?
	Can the antecedents of the behavior of interest be manipulated to change the frequency of the behavior?
Selection consequences	What possible ways does the social/physical ecology reinforce or punish this behavior?
	Does the strength and reinforcement schedule reflect the frequency of the observed behavior?
	Does this type of reinforcement occur in other cultural contexts or geographical settings? If so, do similar behaviors emerge?
	Does the influence of the physical or social ecology outweigh a given individual’s unshared learning history?
	Can the consequences of the behavior of interest be manipulated to change the rate or frequency of the behavior?
Retention antecedents	What would need to change about the physical/social ecology that would precede changes to the behavioral repertoire?
	What features of the social/physical environment preceding the behavior would have to change for the frequency of the behavior to also change?
Retention consequences	What is the adaptive cost of behavior change in the social/physical ecology?
	What consequences of the social/physical environment following the behavior would have to change for the frequency of the behavior to also change?

From there, the remaining questions work to generate a list of potential antecedents and consequences that would contribute to both the selection of a certain behavior (over and above potential alternatives) as well as the retention of that behavior. Among the list of sample questions in Table 1, many fall under the theme of identifying common environmental features occurring in different cultural contexts where similar behaviors are observed. There has been some evidence to suggest that similar contextual contingencies result in similar behaviors across large geographic divides (Henry, 2014). This is likely due to the fact that nowhere in Markus and Hamedani’s (2007) definition of culture is geographic location an essential feature. Culture is multidimensional and spans geographic region through other common features such as religion, socio-economic status, and generation (Cohen, 2014). For example, the exploration of cultural themes of honor in Middle East and southern United States has demonstrated a high degree of similarities as oppose to differences (at least with this particular cultural value; Cohen et al., 1996; Mosquera et al., 2007; Uskul et al., 2015). Examining these particular commonalities may help generate possible antecedents and consequences that work to select and retain this particular cultural value as well as the practices surrounding it.

Likewise, given our understanding of interlocking behavioral contingencies (IBS), it is important to consider *who* or what subgroup of the population is a primary reinforcer of the behavior of interest. What do they gain from reinforcing these behaviors? What other behaviors might they also be motivated to reinforce? It is also possible that some form of mutual reinforcement is involved. Is the person or group of people reinforcing a certain behavior in turn reinforced for their reinforcing behavior? What can this interaction or meta-reinforcement inform us of potential consequences? What other consequences would happen if people within the group stop reinforcing the behavior of interest? Through these near infinite questions, the researcher can begin to develop a list of identified antecedents and consequences that potentially relate to the behavior of interest.

#### Step 2: Determining Functional Relationships

Determining functional relationships between a behavior and its possible contingencies for a cultural group would likely be a similar process as it is for an individual. Essentially, researchers would be looking for a mathematical relationship that would resemble a Bayesian analysis more than a Pearson correlation. Three pieces of information are needed: (1) an overall base rate of a behavior (i.e., cultural practice) occurring in a non-specified setting under non-specified reinforcement contingencies, (2) a list of potential antecedents or consequences occurring in proximity to the behavior (as generated in Step 1), and (3) the probabilities of the behavior occurring before or after a consequence or antecedent, respectively. By comparing the relative base rates of a behavior within a contextual contingency to its overall base rate, a functional relationship can be derived (Sturmey, 1996; Iwata, 2000; Baum, 2017).

For example, imagine that people in both Culture A and Culture B perform Behavior X 25% of the time across all antecedent conditions. However, people in Culture A perform it 85% of the time in situation Y or 100% of the time after experiencing Consequence Z, whereas members of Culture B continue to perform Behavior X at around 25% of the time even in these conditions. This would be an example of a functional relationship between antecedents/consequences and a behavior that varies between culture group. In the case of identifying a differential functional relationship like the one described above, culture membership becomes an additional antecedent condition. There are many statistical methods that could be used to calculate this. A researcher could compare effect sizes, odds ratios, complete a non-parametric analysis, or perform a formal Bayesian analysis.

#### Step 3: Gathering Supporting Evidence

As in a traditional functional behavioral assessment, testing out predictions made by the model in novel situation as well as examining the change in behavior frequency when manipulating the contingencies are essential to supporting the ABC explanation. If the extant evidence has shown that the behavior of interest occurs within one particular context over another, not only should this be replicable within the same culture group, but individuals in other culture groups should perform similarly if the underlying contingencies are manipulated. Likewise, the removal of the specific contingencies that form the functional relationships should reduce the degree to which the behavior is performed within a variety of contexts. Although a wide variety of methodology can be implemented, experimental studies provide an excellent avenue for further elucidating the exact mechanism of reinforcement as well as antecedent-sensitivity.

### Examining Cultural Differences in Social Anxiety Between Japanese and European Americans

In order to provide a case example of this approach, the current set of two studies re-analyze and re-interpret data from the current author’s research program examining cultural differences in social anxiety among people of East Asian Heritage and European Heritage. A brief summary of this field will precede engaging in the three steps outlined above.

Over the past 30 years, the extant research has shown that individuals of East Asian cultural heritage relative to European Americans or European Canadians report higher social anxiety symptoms with remarkable consistency (for meta-analyses see Krieg and Xu, 2015; Woody et al., 2015), with a moderate average effect size of Cohen’s *d* = 0.36 (95%, CI:0.27, 0.44; Krieg and Xu, 2015). Furthermore, follow-up study by Krieg et al. (2018) showed that this cultural difference in social anxiety was not an artifact of non-equivalent measurement properties between the two groups by testing for measurement invariance across common social anxiety measures.

Following the trends in cultural psychology, independent and interdependent self-construal were posited as mediators (Okazaki, 1997; Krieg and Xu, 2015, 2018), where viewing oneself as separate or independent from social others led to reduced social anxiety and viewing oneself as interconnected with social others as either increasing (Okazaki, 1997; Krieg and Xu, 2018) or unrelated to social anxiety (Krieg and Xu, 2015). Employing a cultural neuroscience perspective, self-construal was understood act as a framework for a variety of cognitive functions (Han and Humphreys, 2016), including determining the emotional saliency and relevance to the self, and “threat appraisal” was added to the model (Krieg and Xu, 2018). As originally suggested by Okazaki (1997, 2000), Krieg and Xu (2018) demonstrated that patterns of interdependence (and relatively less independence) transform the perception of a variety of social situations as “high stakes” situations for members of one group more than the other. The results of this process likely contribute to the detection of social threat and subsequent phenomenological experience of social anxiety. An additional study currently in preparation expands the examination of social threat by examining its role as a mediator in a behavior-based, and quasi-experimental study across multiple culture groups.

Although several important questions have already been answered by the extant literature, some compelling questions remain. For instance, what situations are considered ‘socially threatening?’ Do people of East Asian-heritage consider the same situations socially threatening as European-heritage people, or are the two groups responding to different situations? Is expressing social anxiety more adaptive in one context than another? Do differences in the level of adaptability explain cultural group differences in social anxiety? Each of these questions contain elements of a functional relationship between social anxiety and its antecedents (e.g., the situations where it occurs) as well as its consequences (e.g., whether the outcomes are desirable or “adaptive”). Thus, the incorporation of a functional behavioral approach may help in answering these questions.

An important first step of a functional behavioral approach is examining the potential consequences of a certain behavior in general before investigating its function in a particular context. Researchers studying the behavioral ecological ramifications of emotional displays among primates from an evolutionary perspective context that displays of fear or anxiety can function to convey the readiness to submit (Fridlund, 1994), communicate the intention to avoid threat (Keltner, 2003), and request assistance and support from social others (Eisenberg et al., 1989). Taken together, one of the key general functions of displaying social anxiety is to garner social support and sympathy. Although a general function is not always helpful when it comes to understanding a behavior’s function within a specific context, it can be a good place to begin one’s investigation.

There is some supporting evidence for the viability of this universal function in helping explain differences in the expression of social anxiety between groups of Japanese and European Americans. In a recently published preprint that examined Japanese and European American responses to a vignette of someone suffering from social anxiety, the Japanese responses were more positive and neutral, whereas the European American responses were more negative and judgmental (Krieg et al., 2019). The authors concluded displaying social anxiety facilitated a more positive and even sympathetic reaction among Japanese participants, who used words such as “sensitive,” “victimized by own thoughts,” and “tendency to care too much” to describe the character in the vignette as compared to European Americans who used words such as “awkward” and “insecure.” Therefore, it is plausible that social anxiety functions to facilitate social support in a social situation.

### Current Studies

In a contextual behavioral account of culture, social anxiety would not be something “felt,” but rather behaviorally signaled to others. This might be in the form of a fearful expression, stuttered speech, averted eye-gaze, behavioral avoidance, or an endorsement of social anxiety on a questionnaire. All of these signals (among many more) are explicit behavioral attempts to convey social anxiety by the participant to others in their environment. Following the three steps outlined above, we aim to identify potential contingencies, determine functional relationships, and gather supporting evidence to better understand how signaling social anxiety functions between groups of Japanese participants and European-American participants.

Krieg et al. (2018) originally used the self-construal model to explain differences in threat appraisal that would then predict social anxiety. They hypothesized that viewing oneself as less independent and more interdependent from social others would increase the degree to which ambiguous social situations would be perceived as threatening, thereby increasing social anxiety. In contrast, Study 1 re-analyzes situation data collected as a part of Krieg et al. (2018) in order to identify contexts in which social anxiety was signaled differentially by one group over and above the typical culture group differences in social anxiety. We hypothesize that Japanese and European American participants will both be able to generate a number of diverse situations in which they experience social anxiety. We also hypothesize that the two culture groups will respond differently to some situations over and above the patterns of responding identified across the pool of situations. These situations may be able to be organized by the theme of social-support seeking in uncomfortable situations.

Following the results of Study 1, Study 2 simulates experiment data based on descriptive statistics collected from Krieg (2018) and Okazaki et al. (2002) in order to test the tentative conclusions drawn from Study 1. The simulated data will mirror prior findings as well as conform to typical experimental protocols to make it as realistic as possible. All in all, both of the studies are designed to examine the predictive utility of the following functional statement: Signaling social anxiety functions to garner social support or assistance when in a particular situation among Japanese culture group members relative to European-American culture group members.

## Study 1 Methods

### Participants

The sample from Krieg (2018) Study 1 was used to examine social anxiety across antecedent conditions. This sample consisted of 212 Japanese (116 females; *M*_age_ = 20.88; *SD* = 2.23) and 249 European Americans (180 females; *M*_age_ = 21.14; *SD* = 5.01). All participants signed an informed consent, and this study was approved by the University of Hawai‘i at Mānoa’s Human Studies Program (CHS #22337). For more information, please see the original publication.

### Procedure

In order to produce a repository of participant-generated social-anxiety provoking situations (e.g., antecedent conditions), we incorporated a situation sampling approach (Kitayama et al., 1997; Morling et al., 2002). First, a pilot study was first conducted to identify social situations that were perceived by individuals of Japanese and European-American heritage as anxiety provoking. The pilot study recruited 30 Japanese nationals and 30 European Americans. To generate a pool of situations that are relatively salient to members of the three cultural groups in provoking their social anxiety, participants responded to an open-ended question online, “For the following categories, please create brief, specific situations where someone would feel socially anxious,” and generated a total of 610 unique social situations; 313 by Japanese participants and 297 by European American participants.

In accordance with the situation sampling method, each participant in the current sample answered a unique set of randomly selected situations. We randomly selected 30 situations, 15 generated from each cultural group and asked the current participants to rate the degree to which they would experience social anxiety in this situation (“How anxious would you feel if this situation happened to you?”; 1 = None, 5 = Extremely Anxious). Please see Krieg (2018) for a detailed description of situation preparation, cleaning, and redistribution procedures.

## Study 1 Results

### Step 1: Identifying Potential Contingencies

In order to generate a list of antecedent conditions that would provoke social anxiety among members of each culture group, we used the first part of the situation sampling procedure [described as ‘pilot study’ in Krieg’s (2018) Study 1 “Methods” section]. The results of the pilot study were 610 social situations (297 from Europeans American and 313 from Japanese participants).

### Step 2: Determining Functional Relationships

**FIGURE 1:**
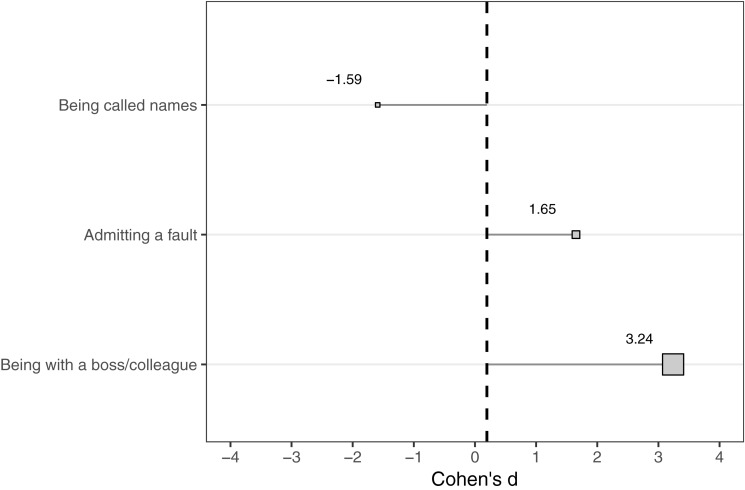
Cohen’s *d* effect sizes of culture group differences in social anxiety under three antecedent conditions relative to overall base rate effect size.

In order to establish a base rate of culture group differences in endorsed social anxiety, we calculated a Cohen’s *d* effect size for the anxiety scores overall, irrespective of each situation. The base rate for the culture group differences in social anxiety was Cohen’s *d* = 0.194 (95% CI: 0.152, 0.236). From this point, we calculated the effect sizes for culture group differences in social anxiety for each situation. The confidence intervals of these effect sizes were compared in order to identify if any situation antecedents produced a culture group difference in social anxiety over and above the base rate. Among all 610 situations, only three situations were associated with effect sizes that were either statistically significantly above or below the base rate and not overlapping with 0 (no effect). These six situations were *being with a boss/colleague* (*d* = 3.24; 95% CI = 0.77, 5.70), *admitting a fault* (*d* = 1.65; 95% CI = 0.30, 3.00), and *being called names* (*d* = −1.59; 95% CI = −2.99, −0.19). All of these situations were made by European-American participants (see Figure 1).

## Study 1 Discussion

When all of the variance from situations were collapsed into a single global category, a small-to-moderate culture group difference in social anxiety endorsements emerged, with Japanese participants scoring higher than European American participants. This particular effect size corresponds with the findings from prior meta-analyses (Krieg and Xu, 2015; Woody et al., 2015). As identified and disseminated in the original Krieg (2018) manuscript, both Japanese and European Americans generated approximately the same number of socially anxiety provoking situations in the first part of the situation sampling analysis. This result suggests that the saliency of social anxiety-provoking situations is approximately the same between the two culture groups. That said, the specific types of situations identified varied widely, with standardized culture group differences in social anxiety varying as a function of these situations. From a contextual behavioral perspective, it seems that the antecedents for social anxiety differ between the two groups, with some antecedents holding more influence on whether social anxiety will be endorsed.

Most situations had effect sizes that were unable to be differentiated from the overall effect, with only three situations being identified as “above and beyond” the base rate. These three situations had effect sizes showing Japanese participants endorsing more social anxiety than European Americans (*n* = 2) and vice versa (*n* = 1). When examining these situations for themes, it became apparent that in the two situations where Japanese participants expressed more social anxiety than European Americans contained elements of “having made a mistake in an official situation” (e.g., admitting a fault and being in a workplace setting). In contrast, the situation where European Americans endorsed more social anxiety when being verbally attacked by another person. By examining the patterns of effect sizes in these “over and above situations,” we gained some preliminary evidence supporting the help-seeking function of social anxiety. Thus, we can generate a functional statement as follows:

Displaying social anxiety [behavior] *functions to* garner social support or assistance [consequence] *when* having made a mistake in an official situation [antecedent] *among* Japanese relative to European Americans.

## Hypothetical Study 2

In order to gather supporting evidence (Step 3) of our proposed functional statement, we conducted a quasi-experiment that directly tests the hypothesis through manipulating antecedent conditions. If the main difference between the two groups in terms of the functional consequences of social anxiety is its amenability to garner social support or assistance when having made a mistake in an official situation, then the degree of social anxiety expressed should vary in conditions were social support is potentially available as compared to social situations where it is less available. Study 2 is comprised of a hypothetical experiment of a speech task designed to elucidate the necessary antecedent conditions for the desired consequences of social anxiety among Japanese as compared to European Americans.

## Study 2 Methods

### Participants

The simulated sample included 200 participants, 100 Japanese (50 female) and 100 European Americans (50 female). The mean age for these participants was 22.95 (*SD* = 2.10) and 21.82 (*SD* = 1.73) for each culture group, respectively.

### Procedure

Participants were tasked with giving a 5-min speech in which they admit to a recent mistake they made at their workplace. After receiving 5 min to prepare, half of the participants were randomly assigned to give their speech in front of a video camera (camera condition) while viewing a video of stock footage of a small classroom listening to a speech projected on a nearby wall. No one else was in the room while the speech was being video recorded. The other half gave their speech in the presence of seven confederates (research assistants matched to the participant’s cultural background). In the audience condition, the speech was also video recorded. Upon completing the speech, participants were escorted to a separate room where they completed questionnaires that measured social performance anxiety and perceived social support. Given that the study’s hypotheses and experimental conditions were concealed at the beginning of the study in order to reduce the impact of response bias. Participants were debriefed of the study’s hypothesis and conditions following the completion of the speech task by the principal investigator.

### Measures

#### Social Performance Anxiety

The Social Phobia Scale Six-Item Version (SPS-6; Peters et al., 2011) is a short version of Mattick and Clarke’s (1998) Social Phobia Scale, and measures social performance anxiety on a 5-point Likert scale (0 = not at all characteristic or true of me; 4 = extremely characteristic or true of me). The six-item version was created by selecting items with the best psychometric properties using Item Response Theory. As a result, the SPS-6 has excellent reliability, validity, and sensitivity to change properties (Peters et al., 2011). Krieg et al. (2019) found that the SPS-6 was scalar invariant between Japanese and European American samples, allowing means to be compared. The inter-item reliability within the current sample is 0.88 for Japanese and 0.93 for European Americans.

#### Perceived Social Support

In addition to measuring self-reported social anxiety, we administered a single-item question to participants asking, “how often did you feel that your audience was supporting you as you made your speech.” Responses were on a 5-point Likert scale with 0 representing “not at all” and 4 representing “all the time.”

#### Social Anxiety Displays

Three bilingual research assistants (two Japanese and one European American) who were blind to experimental conditions assigned to participants, rated each participant’s behavioral displays of anxiety from the video recordings. Specifically, the research assistants used the Behavioral Assessment of Speech Anxiety (BASA; Mulac and Sherman, 1974), a standardized behavioral assessment scale, to rate specific behaviors associated with social anxiety. The BASA examined eighteen specific behaviors, e.g., fidgeting, swallowing, breathing heavy, and each were coded using a 7-point Likert scale (1 = not at all, 7 = strong). Each rater coded all of the videos and inter-rater reliability was calculated as an intra-class correlation of 0.91 (CI: 0.88, 0.94). Final scores consisted of the rounded average. All eighteen ratings were summed together to generate a final behavioral score (alpha = 0.83). The BASA has demonstrated evidence of internal consistency, inter-rater reliability, and concurrent validity with expert ratings of speech performance in prior Western studies (Mulac and Sherman, 1974; Heeren et al., 2012). Simulated means and standard deviations for each group can be found in Table 2.

**TABLE 2:** Means and standard deviations of simulated SPS-6, social support, and BASA data for each culture group and condition.

		Japanese (*n* = 100)		European Americans (*n* = 100)	
Variable	Condition	Mean	*SD*	Mean	*SD*
SPS-6	Camera	3.33	0.76	2.98	0.89
	Audience	3.48	0.66	2.69	0.85
	Overall	3.41	0.71	2.84	0.88
Social support	Camera	1.90	0.57	1.97	0.51
	Audience	2.33	0.39	0.205	0.33
	Overall	2.12	0.54	2.01	0.42
BASA	Camera	32.28	6.74	34.11	6.05
	Audience	37.53	5.53	34.86	6.41
	Overall	34.90	6.68	34.49	6.21

*SPS-6, Social Phobia Scale – Six item version; BASA, Behavioral Assessment of Speech Anxiety.*

## Study 2 Results

### Step 3: Gathering Supporting Evidence

#### Mean Differences

In order to gather additional support hypothesis that increased displays (endorsements) of social anxiety functioned primarily in situations where having made a mistake in an important situation, we first explored mean differences between culture groups and experimental conditions. To this end, we implemented a multivariate general linear model that regressed social performance anxiety, perceived social support, and social anxiety displays on culture group, experimental condition, and the interaction between the two.

For social performance anxiety, as measured by the SPS-6, our analysis revealed a statistically significant main effect for culture group (*B* = 0.34, *p* = 0.033), with Japanese participants reporting experiencing more social anxiety. There was no effect found for experimental condition (*B* = −0.29, *p* = 0.72), and the results of the interaction effect were also statistical significance (*B* = 0.45, *p* = 0.049).

For perceived social support, our results demonstrated no main effect for culture group (*B* = −0.07, *p* = 0.442), or experimental condition (*B* = 0.08, *p* = 383). However, the culture group x experimental condition interaction effect was also statistically significant (*B* = 0.35, *p* < 0.01), with Japanese participants scoring higher overall, but especially in the live audience condition.

**FIGURE 2:**
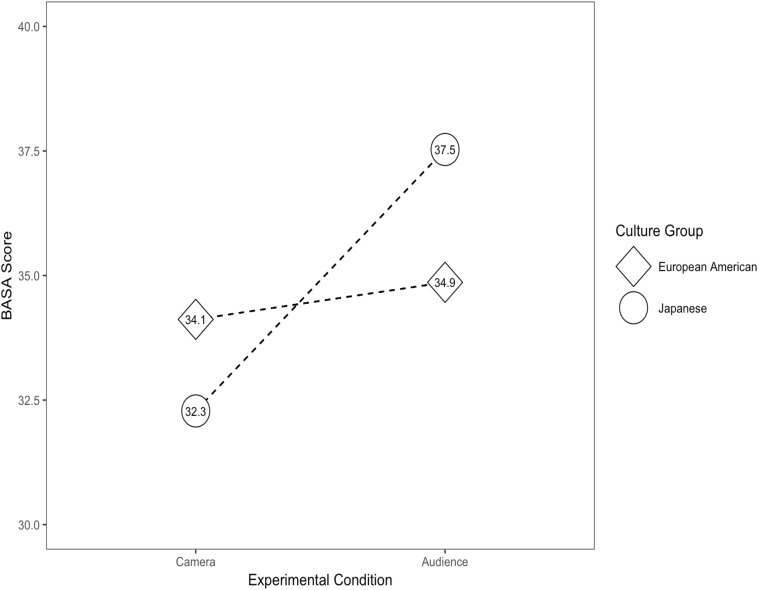
Results of hypothetical study depicting the culture group × experimental condition interaction’s influence on social anxiety scores. BASA, Behavioral Assessment of Speech Anxiety.

Finally, for social anxiety displays, as measured by the BASA, our analysis revealed no main effect for either group (*B* = −1.83, *p* = 0.140) or experimental condition (*B* = 0.74, *p* = 0.550). However, the results did demonstrate a statistically significant interaction effect for culture group × experimental condition (*B* = 4.50, *p* = 0.011), with Japanese participants scoring higher in the live audience condition (see Figure 2).

#### Mediation Modeling

Given that the pattern of mean differences suggested that the experimental condition may predict social anxiety displays and perceived social support among Japanese but not necessarily European Americans, we used structural equation modeling (SEM) to examine the potential mediating effect of social anxiety displays in the relationship between experimental condition and perceived social support.

Specifically, we constructed two path models, one for Japanese participants and one for European American participants. We specified direct paths from experimental condition (1 = live audience) and perceived social support, as well as between perceived social support and anxiety displays. Social performance anxiety was included as social anxiety display’s covariate. Each model was estimated using the maximum likelihood estimator implemented with the ‘lavaan’ package (Rosseel, 2012) with R (R Core Team, 2014).

**FIGURE 3:**
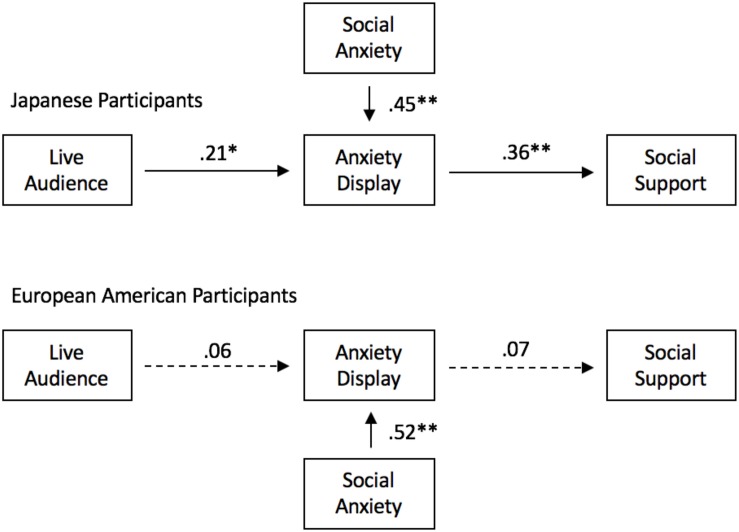
Hypothetical path model and standardized coefficients for each cultural group. ^∗^*p* < 0.05 and ^∗∗^*p* < 0.01.

The results of our analyses revealed that among Japanese participants, social anxiety displays fully mediated the relationship between experiment condition and perceived social support (CFI: 0.999, TLI: 0.998, RMSEA: 0.036, and SRMR: 0.023). However, for European Americans, neither path coefficient was statistically significant, and model fit was low (CFI: 0.818, TLI: 0.453, RMSEA: 0.421, and SRMR: 0.192). See Figure 3 for a depiction of the model as well as specific details on path coefficients.

## Study 2 Discussion

The results of Study 2 demonstrate the impact of the antecedent situation on the cultural group differences in social anxiety. By changing one aspect of the antecedent (i.e., camera vs. live audience) the behavioral expression changed based on its intended function. Assuming that the hypothetical experiment replicates with real experimental data, Study 2 would also provide the additional evidence needed to support the functional statement derived from Study 1. We would have provided support for the functional statement, “displaying social anxiety functions to garner social support or assistance when having made a mistake in an official situation among Japanese relative to European Americans.” However, it is also important to note that behavior can have multiple functions within the same context. Because anxiety displays can function to foster social support in one group does not necessarily mean that it cannot also be a reaction to the threat of being scrutinized or criticized by peers as concluded in Okazaki (1997) and Krieg et al. (2018).

That being said, this functional statement is fundamentally different than statements previously made in the literature that associated social anxiety behavior with mentalistic constructs like independent and interdependent self-construal in that each aspect can be directly observed and requires no further explanation (e.g., “where does self-construal come from,” “where is it located,” “is it malleable across the lifespan,” and “what predicts self-construal,” etc.). It also positions culture in a larger context that is not limited to geography or nationality. For instance, if social anxiety functions to facilitate social support among Japanese people, are there other groups that seek social support in a similar way? What about a different way? Would the different group’s social support seeking strategies also work in a Japanese context? If not, what alternative consequences are generated instead? Have social support seeking strategies changed over time? Does this align with changing contingencies in the environment or simply a product of behavioral variation (e.g., cultural drift)? Many of these questions can be asked and answered within single sample studies.

Furthermore, examining social anxiety through a CBS lens centers the examination on adaptability rather than pathology, reducing the stigma of certain behaviors in certain groups by explicitly stating how the behavior is reinforced by desirable consequences (or avoiding undesirable consequences.). This hypothetical study would posit a new conclusion is that social anxiety is more adaptive in a Japanese context than an European American context in terms of garnering social support, which is a very different conclusion than suggesting that people of Japanese heritage being intrinsically more anxious than European Americans. The concept of differential adaptability is necessarily focused on the environment, rather than the person or group of people responding to it.

## General Discussion

In the current studies, we attempted to provide a simple example of implementing functional behavioral methodology in an established program of culture research. First, we generated a list of antecedent conditions and proposed functional consequences from the extant literature (Step 1). Then we determined functional relationships by examining differences in the effect sizes of a behavior among different antecedents relative to its overall base rate (Step 2). We then used a functional statement to generate testable hypotheses and explicitly sought evidence to support the statement (Step 3).

It is important to note that there are already many cultural psychologists doing work that would be proposed by this model, though maybe under a different name. For instance, the socioecological framework also emphasizes antecedent conditions within a social ecology to predict differences in cultural practices (Talhelm and Oishi, 2019). For instance, Gelfand et al. (2011) examined the degree to which tight situational constraints in modern cultures could be explained by historical population density. Arguing that historical population density created a survival pressure in these cultures, their study showed a strong correlation between ecological antecedents and cultural behaviors. Their conclusion, however, is more of an explanatory statement (answering “why”) than a functional statement (answering “what for”).

Some advantages of a CBS perspective include the emphasis on adapting to one’s context (de-pathologization), focus on directly measurable behaviors (observable behavioral outcomes), and the amenability to intervention development. By describing how certain behaviors “work” in a certain context, but not necessarily others, the emphasis shifts from pathology to adaptability. The idea of differential adaptability is especially important in potentially explaining functional differences in behavior or a behavioral syndrome between groups in a non-stigmatizing manner.

Likewise, by having behavioral definitions for each construct of interest, we reduce the challenges associated with relying on mentalistic concepts (Ryle, 1984; Baum, 2017). Given that the main objective of CBS is to predict behavior and successfully intervene based on its function, once we have established and supported a functional statement, further hypotheses abound. We could expand the statement to include multiple consequences or a series of antecedents in order to better predict behavior in context as well as design effective interventions.

Adapting culture-group findings to individual clients is not terribly difficult. With this type of analysis already in place, updating the model to include information related to a client’s idiographic learning history is entirely plausible. A clinician can examine the degree to which known contingencies associated with a given behavior apply to their client and can structure questions and in-session activities to gather further evidence. Sharing functional analyses with clients can also be helpful in facilitating a new understanding on how concerning behavior is reinforced and may have been initially or occasionally adaptive.

No approach is without its limitations, however, and one important limitation is that behavior analysis itself is a culture (Ruiz and Roche, 2007), and using behavioral language evokes a set of Western values associated with action, causation, health, and wellbeing. By emphasizing these constructs, there is the possibility of missing important information that doesn’t necessarily conform to a behavioral framework. Likewise, although in clinical practice, behavior analysts are focused on the ideographic saliency of a reinforcer for each client, that nuance seems to be lost when using the same model to compare behaviors among different culture groups. To this point, there is evidence to suggest that, in general, European Americans find contingencies related to enhancing one’s influence on others more salient, whereas Japanese participants were more focused on contingencies that highlighted adjustment to others (Morling et al., 2002). Not every antecedent or consequence can be readily compared. Keeping these potential limitations in mind, cultural scientists or culture-clinical researchers can evoke the strengths of this approach to better understand culture’s influence on behavior in context.

## Data Availability Statement

The raw data supporting the conclusions of this article will be made available by the authors, without undue reservation, to any qualified researcher.

## Ethics Statement

The studies involving human participants were reviewed and approved by University of Hawaii Human Studies Program. The patients/participants provided their written informed consent to participate in this study.

## Author Contributions

The author confirms being the sole contributor of this work and has approved it for publication.

## Conflict of Interest

The authors declares that the research was conducted in the absence of any commercial or financial relationships that could be construed as a potential conflict of interest.
